# Journal of Hematology & Oncology reviewer acknowledgement 2012

**DOI:** 10.1186/1756-8722-6-20

**Published:** 2013-03-28

**Authors:** Delong Liu

**Affiliations:** 1 Westchester Medical Center, Munger Pavilion 250, Valhalla, NY 10595, USA

## Abstract

**Contributing reviewers:**

The editor of *Journal of Hematology & Oncology* would like to thank all our reviewers who have contributed to the journal in Volume 5 (2012).

## 

Yasuhito Abe

Japan

Lawrence Afrin

United States of America

Haidar Akl

Belgium

Haifa Kathrin Al-Ali

Germany

Ina Amarillo

United States of America

Borje Andersson

United States of America

Kelly Andringa

United States of America

Emmanuel Antonarakis

United States of America

Thomas Aparicio

France

Fumio Arai

Japan

David Araten

United States of America

Jane Armstrong

United Kingdom

Ulrike Bacher

Germany

Olga Balague

Netherlands

Sanjay Bansal

United States of America

Frederic Baron

Belgium

Giovanni Barosi

Italy

John Barrett

United States of America

Surinder Batra

United States of America

Paul Beckett

United Kingdom

Tanios Bekaii-Saab

United States of America

Asim F. Belgaumi

Saudi Arabia

Francis Belloc

France

Frank Berger

Germany

Olivier Bernard

France

Didier Bouscary

France

Massimo Breccia

Italy

Ansgar Brüning

Germany

Alan Burnett

United Kingdom

Gianni Cazzaniga

Italy

Jan Cerny

United States of America

Xiu-bao Chang

United States of America

David Z Chang

United States of America

Nelson Chao

United States of America

Mingyi Chen

United States of America

Jen-Shi Chen

Taiwan

Yeu-Chin Chen

Taiwan

liguang chen

United States of America

Jen-Yang Chen

Taiwan

Chong Chen

United States of America

CS Chim

China

Bertrand Coiffier

France

Enrique Colado

Spain

Brian Curtis

United States of America

Zunyan Dai

United States of America

Maria Delioukina

United States of America

Enrico Derenzini

Italy

Arjan Diepstra

Netherlands

Joshua Doloff

United States of America

Jia-Hong Dong

China

Jeanette Doorduijn

Netherlands

Elias Drakos

Greece

Marc Earl

United States of America

Thomas Efferth

Germany

Avraham Eisbruch

United States of America

Andreas Eisenreich

Germany

Evgeniy Eruslanov

United States of America

Emiliano Fabiani

Italy

Franca Fagioli

Italy

Hugo Fernandez

United States of America

William Figg

United States of America

Alessandro Fornari

Italy

Claudio Fozza

Italy

Hui Fu

United States of America

Seiji Fukuda

Japan

Muhammad Furqan

United States of America

Apar Kishor Ganti

United States of America

Javid Gaziev

Italy

Ulrich Germing

Germany

Romi Ghose

United States of America

Swati Goel

United States of America

Gerard Grosveld

United States of America

Jian Guan

United States of America

Milena Gusella

Italy

Claudia Haferlach

Germany

Anne M.M.J. Hagemeijer

Belgium

Jean-Luc Harousseau

France

Moustapha Hassan

Sweden

Shigetsugu Hatakeyama

Japan

Georgia Hatzivassiliou

United States of America

Grzegorz Helbig

Poland

Nobuko Hijiya

United States of America

Heather Himburg

United States of America

Jessica Hochberg

United States of America

Akira Horikoshi

Japan

Katsuyuki Hotta

Japan

Jian Hou

China

Chung-Tsen Hsueh

United States of America

Zhenbo Hu

United States of America

Xi Chun Hu

China

Ruoqing Huang

United States of America

Xuan Huang

United States of America

Yao Huang

United States of America

Tiangui Huang

United States of America

klaudia hunter

United States of America

Yasuo Imai

Japan

Esma Isenovic

United States of America

Martin Jädersten

Sweden

Siegfried Janz

United States of America

Elizabeth Jeffery

United States of America

David Johnson

United Kingdom

Edgar Jost

Germany

Lawrence Jung

United States of America

Marketa Kalinova

Czech Republic

Rotem Karni

Israel

Friederike Keating

United States of America

Santosh Kesari

United States of America

Won Seog Kim

Korea South

Shinya Kimura

Japan

Ulf Klein

United States of America

Dong Hoe Koo

Korea South

Ioannis Kotsianidis

Greece

Ralf Kueppers

Germany

Naoki Kunii

Japan

Mei-Ling Kuo

United States of America

Andrey Kuznetsov

Austria

Yok Lam Kwong

China

Michael La Quaglia

United States of America

Jatinder Lamba

United States of America

Gurpreet Lamba

United States of America

Richard Larson

United States of America

Tahir Latif

United States of America

Ulrich Lehmann

Germany

Carlos Leon

Canada

Yangqiu Li

China

Lei Li

United States of America

David Linch

United Kingdom

Qi-Fa Liu

China

Bei Liu

United States of America

Zach liu

United States of America

Paolo Lunghi

Italy

Yupo Ma

United States of America

Nicol Macpherson

Canada

Ignazio Majolino

Italy

Esther Manor

Israel

Maurizio Margaglione

Italy

Alberto Maria Martelli

Italy

Giovanni Martinelli

Italy

Olivia Martinez

United States of America

Melanie McConnell

New Zealand

Jason Merker

United States of America

Yutaka Midorikawa

Japan

Joseph Mikhael

United States of America

Peppino Mirabelli

Italy

Roberto Miranda

United States of America

Jun Miyauchi

Japan

Yasushi Miyazaki

Japan

Hiroyuki Momota

Japan

Patrick S. Moore

United States of America

Michael Morse

United States of America

Toru Motokura

Japan

Marek Mraz

Czech Republic

Arend Mulder

Netherlands

Antonino Musolino

Italy

Marta Muzio

Italy

Kyriaki Mystakidou

Greece

Yona Nadir

Israel

Shotaro Nakamura

Japan

Steffan Nawrocki

United States of America

Gustav Nilsonne

Sweden

Maria D. Odero

Spain

Shouichi Ohga

Japan

Takayuki Ohguri

Japan

Seiichi Okabe

Japan

Barry O'Keefe

United States of America

Yasuhiro Oki

United States of America

Nicolas Ortonne

France

Giuseppe Palumbo

Italy

Ping-Ying Pan

United States of America

Jen-Jung Pan

United States of America

Sun Park

Korea South

In Hae Park

Korea South

Wendy Parker

Australia

Steven Pavletic

United States of America

Marc Peeters

Belgium

Alex Philchenkov

Ukraine

Pier Paolo Piccaluga

Italy

Josef Prchal

United States of America

Christoph Röllig

Germany

Peter Reimer

Germany

Ruibao Ren

United States of America

Thomas Renne

Sweden

Carmelo Rizzari

Italy

Vivek Roy

United States of America

Soya Sam

United States of America

Birgitta Sander

Sweden

Rafael Santana Davila

United States of America

Daniele Santini

Italy

Marit Saunes

Norway

Akira Sawaki

Japan

Peter Sayeski

United States of America

Devin Schellenberg

Canada

Hideharu Sekine

Japan

Lijian Shao

United States of America

Nima Sharifi

United States of America

Aubie Shaw

United States of America

Changxian Shen

United States of America

Adawiyah Suriza Shuib

Malaysia

Said Sif

United States of America

Nicola Silvestris

Italy

Susan Slovin

United States of America

Giedre Smailyte

Lithuania

Eric Solary

France

Wenru Song

United States of America

Martin Sos

United States of America

Wolfgang Sperr

Austria

Keith Stewart

United States of America

Jan Styczynski

Poland

Weijing Sun

United States of America

Kazutaka Sunami

Japan

Monalisa Sur

Canada

Julien Taieb

France

Jerome Tamburini

France

Angela Tan

Australia

Susan Thorpe

United Kingdom

Kensei Tobinai

Japan

Mauro Tognon

Italy

Tomomi Toubai

United States of America

Adrian Trifa

Romania

Ching-Hwa Tsai

Taiwan

William Tse

United States of America

Saad Usmani

United States of America

Remco van Doorn

Netherlands

Amit Verma

United States of America

Luis Villela

Mexico

Edmund Waller

United States of America

Naomi Walsh

Ireland

Jianxiang Wang

China

Sa Wang

United States of America

Hongxia Wang

China

Jing Wang

China

Guoqing Wei

China

JoEllen Welsh

United States of America

Meir Wetzler

United States of America

Alice Wong

Hong Kong

David Wong

United States of America

Nina Worel

Austria

Kongming Wu

United States of America

Jie Wu

United States of America

Yu-Chung Wu

Taiwan

Yong-Bing Xiang

China

Kang Xiaonan

China

Dazhong Xu

United States of America

Zhi Xu

United States of America

Mingjiang Xu

United States of America

Yutaka Yamamoto

Japan

Takahiro Yamauchi

Japan

Masamitsu Yanada

Japan

Xiaoming Yang

United States of America

Yibin Yang

United States of America

David Yang

United States of America

Xiao-Feng Yang

United States of America

Yong-Gang Yao

China

Jeffrey Ye

United States of America

Chih-Ching Yeh

Taiwan

Tzu-Chen Yen

Taiwan

Jian Yu

United States of America

Bo Yuan

Japan

Yuan Yuan

United States of America

Zhao-Chong Zeng

China

Hongyong Zhang

United States of America

Hua Zhang

China

Zhen Zhao

United States of America

Zhizhuang Joe Zhao

United States of America

Xianfeng Zhao

United States of America

Xiaoyong Zheng

United States of America

Yunli Zhou

United States of America

Ping Zhu

China

Pier Luigi Zinzani

Italy

Axel zur Hausen

Netherlands

